# Investigation of a 3D system distortion correction method for MR images

**DOI:** 10.1120/jacmp.v11i1.2961

**Published:** 2010-01-28

**Authors:** Teodor Stanescu, Hans‐Sonke Jans, Keith Wachowicz, B. Gino Fallone

**Affiliations:** ^1^ Medical Physics Cross Cancer Institute Edmonton AB Canada

**Keywords:** MRI, distortion correction

## Abstract

Interest has been growing in recent years in the development of radiation treatment planning (RTP) techniques based solely on magnetic resonance (MR) images. However, it is recognized that MR images suffer from scanner‐related and object‐induced distortions that may lead to an incorrect placement of anatomical structures. This subsequently may result in reduced accuracy in delivering treatment dose fractions in RTP. To accomplish the accurate representation of anatomical targets required by RTP, distortions must be mapped and the images rectified before being used in the clinical process. In this work, we investigate a novel, phantom‐based method that determines and corrects for 3D system‐related distortions. The algorithm consists of two key components: an adaptive control point identification and registration tool and an iterative method that finds the best estimate of 3D distortion. It was found that the 3D distortions were successfully mapped to within the voxel resolution of the raw data for a $260\times 260\times 240\;{\text{mm}}^{3}$ volume.

PACS numbers: 87.61.‐c, 87.53.Tf, 87.53.Xd, 87.56.‐v, 87.56.Fc, 87.62.+n

## I. INTRODUCTION

One of many applications of magnetic resonance imaging (MRI) is in radiation treatment planning (RTP) of cancer sites. RTP requires precise information about the shape and location of the tumor and the structures‐at‐risk to ensure accurate delivery of radiation to the patient. Due to its remarkable soft‐tissue contrast, MRI has proven to be the preferred imaging modality for the segmentation of anatomical structures. However, it is recognized that MR images suffer from system‐related and patient‐induced distortions that alter the accurate representation of anatomical structures (i.e. spatial location and relative intensity). The system‐related distortions are mainly generated by inhomogeneities in the main magnetic field and non‐linearities in the applied magnetic field gradients that are inherent to any MRI scanner. In contrast, the patient‐induced artifacts are produced by susceptibility and chemical shift variations in the imaged sample. Both types of distortions are dependent on the operation conditions (i.e. imaging sequence) and ideally would be corrected before the MR images are integrated into the treatment planning process.

The current work is motivated by our interest in developing 3T MRI‐based radiation treatment planning procedures (i.e. MRI simulation, in particular for brain).^(^ ^1^ ^–^ ^2^ ^)^ The major limitations of MR images in this regard are the lack of electron density information and reduced spatial accuracy due to distortions. However, recent studies showed that brain^(^ ^1^ ^,^ ^2^ ^)^ and prostate^(^ ^3^ ^,^ ^4^ ^)^ MR images can, for the purpose of dose RTP calculations, be converted into CT‐like images by assigning relevant electron density information to structure contours. In addition, the spatial accuracy of the patient MR images is a conditio sine qua non, as the treatment planning process will rely only on the information derived from these images. To be used for MRI simulation, the MR datasets have to be corrected to reduce distortions to a degree that is tolerated in RTP (i.e. spatial accuracy of better than 2 mm).^(5)^

A considerable amount of literature published over the last two decades addresses the mechanism of MR distortions and methods to correct the resulting MR image artifacts.^(^ ^6^ ^–^ ^8^ ^)^ However, in the last few years, there has been an increased interest in developing advanced 3D distortion correction methods that allow an improved control of distortions. Wang et al.^(9)^ investigated a method that relies on Prewitt operators by using a grid sheets phantom filled with a water‐based solution. Doran et al.^(10)^ used a rod‐type phantom built from three orthogonal interpenetrating arrays of water‐filled tubes to assess distortion by analyzing data obtained from three orthogonal planes. Baldwin et al.^(11)^ investigated system‐related distortions by applying the Wang et al. technique and the object‐induced artifacts by using a method based on the work of Bhangwandien et al.^(12)^ The main rational for developing and implementing image distortion correction techniques is that often the manufacturers do not provide comprehensive tools for evaluating and correcting the distortions.^(^ ^9^ ^,^ ^10^ ^)^ A discussion on this issue is presented in Doran et al.

Any distortion correction method relies on two main components: (a) a technique for the identification and registration of the world (reference dataset, e.g. CT) and image (MR dataset) coordinates of control points, and (b) a procedure to determine the distortion field along with an image distortion correction algorithm. Finding the accurate location of both world and image coordinates of the control points is vital as the distortion field is given by the displacement vectors between these coordinates. The determination of control points for registration is not a simple task. Doran et al. generated manually the reference 3D matrix of CT control points – a work‐intensive task for routine quality assurance, especially when using a large number of control points (approx. 10,000) in order to maximize accuracy. Furthermore, manual identification of the control points can introduce additional user‐related errors. Although Wang et al. did not mention the methods used for extracting the world coordinates of the control points and the registration process of these points with the corresponding MR points, we consider them important and describe our approach in detail (see Section II). Due to possible manufacturing imperfections in the grid sheets (see Section II), the representation of control points coordinates might be altered, leading to an incorrect determination of the 3D distortion field.

Another important aspect of the control points’ identification process is related to the innate properties of 3D MR image datasets. It is recognized that the MR images suffer from various image intensity‐related artifacts such as inter‐ and intra‐slice intensity variations (i.e. smooth intensity variation across the volume^(^ ^13^ ^–^ ^15^ ^)^) that can alter the ability to accurately identify the control points’ location using a computer algorithm. Ideally, these image artifacts need to be corrected before the images are analyzed in the distortion evaluation process. In particular, Wang et al. used the same intensity threshold to process images obtained by determining the first derivative along z‐axis and then convolved to a cross mask. Doran et al. also applied a single intensity level to isolate spots on a 2D grid – structures that embed the location of the control points. The use of a unique image intensity threshold might not be suitable for fast and accurate automatic extraction of the control point coordinates due to inherent spatial variation of MR signal intensity. This signal fluctuation is due to $B_{1}$ inhomogeneities caused by receiver/transmitter coil sensitivity variations, and its magnitude is dependent on the imaging sequence.

Doran et al. determined the distortion of each control point along the main axes as the average value of the distortions measured from two orthogonal datasets. This approach represents the first order approximation of the 3D distortion field. Techniques that take into account the mutual interaction among the distortion values along each axis are investigated in this work (see Section II.D).

This work describes a novel and robust phantom‐based method for determining and correcting 3D system‐related distortions of MR images. Our method overcomes some of the limitations of previously published works^(^ ^9^ ^,^ ^10^ ^)^ by introducing: (a) an adaptive method for the accurate and automatic determination of the control points by compensating for MR image intensity inhomogeneities, (b) a polynomial fitting‐based data cleaning tool that facilitates the automatic registration of the CT and MR control points, and (c) an iteration process required to determine the 3D distortion field in the case of methods^(10)^ that rely on the analysis of multiple orthogonal 2D distortion datasets. The algorithm consists of the following main steps: (a) identification, extraction, and registration of the CT (world coordinates) and MR (image coordinates) control points, and (b) determination of the 3D distortion field by using an iterative process along with an image correction technique.

## II. MATERIALS AND METHODS

**Figure 1:**
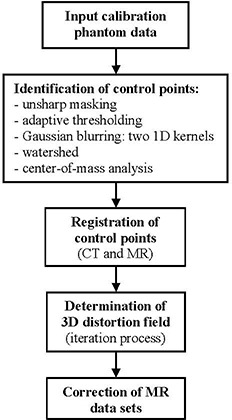
Flowchart of the distortion correction method.

Figure 1 presents the flowchart of our distortion correction method. First, phantom data is acquired and input into the algorithm. Then the MR and CT control points are independently identified by applying a set of image processing steps. The two sets of reference points are registered and the distortion field is determined by using an iteration technique. Finally, individual images are corrected based on distortion maps.

### A. MR/CT phantom

**Figure 2:**
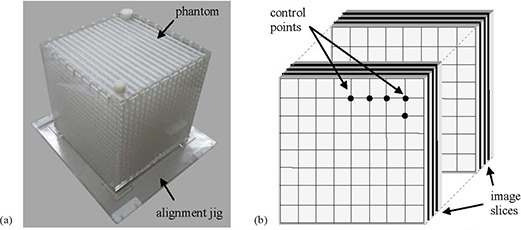
Image sample and diagram depicting the phantom's structure: (a) grid sheet‐based phantom; (b) sample of two subsequent grid sheets separated by a mineral oil gap showing sample control points (the intersection of the grid crosses with the imaged plane and image slices).

We used the same phantom as per Baldwin et al., which was built in‐house similar to that of Wang et al. with slightly modified dimensions (see Fig. 2(a)). Specifically, 17 styrene grid sheets were placed inside a $30\times 30\times 30\;{\text{cm}}^{3}$ empty box with 1 cm thick Perspex walls. The grid sheets are equally distributed inside the box with a spacing of 0.9 cm. Each grid sheet is 0.76 cm thick and contains 17×17 grid points. The spacing between control points is 1.5 cm along each of the two axes in the grid profile plane. The control points, essential for determining the 3D distortion field, are defined by the intersection of the grid crosses with the imaged plane (see Fig. 2(b)). The effective volume covered by the control points is $260\times 260\times 280\;{\text{mm}}^{3}$. To accurately place the phantom in the scanner bore, we built an alignment jig that would minimize positioning errors (≤1 mm) between subsequent scans (see Fig. 2(a)). The jig is made out of Perspex and consists of a plate and four alignment corners inside which the phantom is tightly mounted. Although we used the grid sheet‐based phantom design, our distortion correction method is different than the one developed by Wang et al. (see below). Specifically, we used a combination of adaptive image thresholding along with an iteration technique to determine the 3D distortion field. In contrast, Wang et al. and Baldwin et al. used Prewitt operators to determine the distortion along the main axes. Our procedure can easily be implemented for a rod‐type phantom, too.

### B. Data acquisition

#### B.1 MR scanning procedure

The phantom was filled with mineral oil and scanned on a 3 T Intera (Philips Medical Systems, Cleveland, OH) scanner by using a slightly altered (i.e. larger field of view (FOV)) 3D T1 TFE clinical protocol used for diagnostic and treatment planning of glioblastoma multiforme (GBM) patients. The sequence parameters are as follows: TE/TR/α 4.1 ms/8.8 ms/8°, FOV of $450\times 450\;{\text{mm}}^{2}$ scanned on a 512×512 matrix in‐plane, with 251 partitions, each 1 mm thick and no gap. Once the phantom is mounted on the jig and placed inside the MRI bore, the center of the phantom is located near the isocenter of the scanner. Our control points’ identification method requires that the phantom has 3D symmetry (see below) – that is to say, similar structures are displayed along main axes. However, the grid‐sheet phantom displays control points only in two dimensions (i.e. the intersections of grid lines). The dimension orthogonal to the grid sheet does not offer control points that are easy to evaluate. Rather, the intersection of grid lines occurs in the dimension orthogonal to the grid sheets for the whole width of one grid sheet (0.76 cm). Therefore, distortion values along the dimension orthogonal to the grid plane were obtained by acquiring two more MR axial scans using exactly the same sequence but with the grid plane oriented in a different orthogonal direction each time. Specifically, the phantom was rotated with the grid profile in the desired plane before being mounted on the jig. In total, the phantom was imaged three times and was positioned in such a way that the grid profile was parallel to the transverse (x,y), sagittal (y,z), and coronal (x,z) planes, respectively. The scanners’ software was used to reconstruct the datasets and visualize the grid structures in the respective planes. The same datasets were acquired again but with reversed read gradient to account for residual inhomogeneities in the main magnetic field $\left(B_{0}\right)$.

#### B.2 CT scanning procedure

For the purposes of this paper, CT scans were assumed to be distortion free. A recent study^(16)^ stressed that the distortions in CT images are usually smaller than 1 mm. During a phantom check CT scan, it was observed that some of the grid sheets were significantly warped along the axis orthogonal to the grid profile. This is due to manufacturing imperfections in the commercially available grid sheets. The world coordinates of the control points were therefore obtained from the CT scans, rather than on the nominal design specification of the phantom,^(9)^ which could have some construction errors. The phantom was scanned on a Philips Gemini PET/CT scanner (Philips Medical Systems, Cleveland, OH) in the slice‐by‐slice mode with a $370\times 370\;{\text{mm}}^{2}\;\text{FOV}$, 300 partitions of 1 mm thick each and no gap. The transverse 3D CT scan was reformatted to generate three datasets that would correspond to each of the MR datasets.

### C. CT and MR control points identification and registration

MR images suffer from various image intensity‐related artifacts (e.g. inter‐slice intensity variations and smooth intensity variation across the volume^(^ ^13^ ^–^ ^15^ ^)^) that affect the accuracy of automatically identifying the control points’ coordinates. To address these issues we developed a technique based on a combination of an unsharp masking step with adaptive thresholding that is individually applied to each image (slice) in the 3D dataset. Once the images are preprocessed to account for image intensity‐related artifacts, the control points’ coordinates are given by the center of mass of each area located at the intersection of the grid lines, which are determined by applying 1D Gaussian blurring kernels along the horizontal and vertical axis, respectively. The software was developed in MATLAB (The MathWorks, Inc., Natick, MA) and consists of the following steps (see also flowchart in Fig. 1):

(a) The pixel intensities in the MR images only are inverted in order to display a bright grid profile on dark background, similar to the CT images. Therefore, the same analysis can be applied for both MR and CT images;
(b) An unsharp masking process, which subtracts a strongly Gaussian blurred version of the image from the original one. This step removes low frequency spatial information from the images, highlighting the structures of interest (i.e. the grid pattern);
(c) An adaptive pixel intensity threshold process based on each image histogram is automatically determined (median) to account for intensity variations across the 3D MR volume. The threshold value was set low enough to depict the entire grid structure but sufficiently high to remove background noise;
(d) Once a threshold is selected, two separate image masks are generated: one by applying a 1D Gaussian blurring kernel along the horizontal axis and the other as a result of a similar 1D Gaussian blurring kernel applied along the vertical axis. The two images are summed and only the high intensity pixel areas defined by the intersection of the orthogonal lines are kept, resulting in a new image which contains dots at the intersection of the horizontal and vertical lines;
(e) The dots embed the relative coordinates of the control points; they are separated using a watershed transform (MATLAB) and analyzed individually;
(f) Areas that contain significantly more or less pixels than predefined limits – number of pixels corresponding to a typical dot embedding a control point (e.g. 15) – are discarded as artifacts. This step is particularly important for the CT datasets, which shows the phantom frame and the scanner couch;
(g) The coordinates of each control point are determined from the center of mass of each dot area (defined at step d);
(h) As an optional step, the user can visually inspect the accuracy of the algorithm's output, namely the control points can be overlaid on the MR and CT raw images;
(i) Relative coordinates of the MR and CT control points are stored and used for registration and 3D distortion field analysis;
(j) For the registration process, the origin was set to be at the isocenter of the MR scanner. The registration of the CT to MR datasets can be described as a two‐step process. First, the CT slice corresponding to slice z=0 in the MR dataset is identified by determining the z dimension of the CT scan (and/or using MR‐CT compatible fiducial markers, such as copper sulphate). Secondly, the *x* and *y* shifts are determined by identifying on the CT central slice (z=0) the control point corresponding to the MR control point located at the isocenter and by calculating the displacement value between the two. In this process, it was assumed that the magnitude of distortion is negligible near the isocenter of the scanner, a valid premise in the field.^(10)^

**Figure 3:**
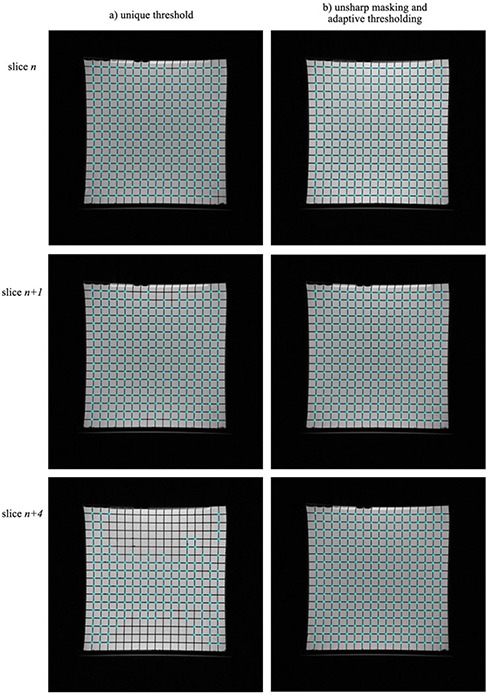
Comparison of two techniques used to identify and extract the MR control points: (a) unique threshold for all images ‐ the threshold was set on slice *n* and used subsequently for slices n+1 and n+4; (b) unsharp masking and adaptive thresholding applied to each image.

**Figure 4:**
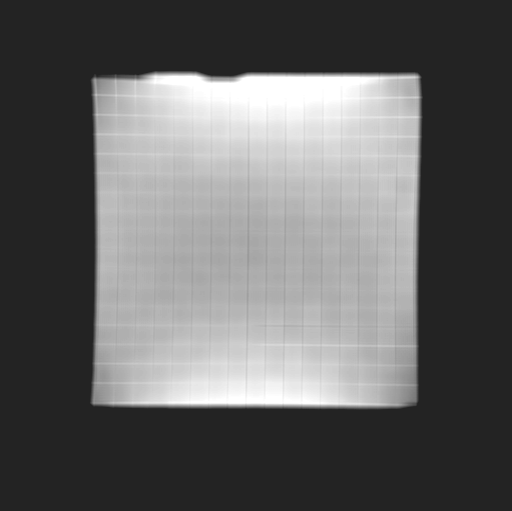
Example showing typical image intensity inhomogeneities removed from MR raw images by applying unsharp masking.

The importance of the unsharp masking and adaptive thresholding processing (b and c, above), is stressed by the two sets of image slices displayed in Fig. 3. The columns correspond to images processed by using a unique threshold and by applying steps (b) and (c), respectively. The unique threshold was initially set on the first image (slice *n*), where control points are readily identified by the algorithm (two control points were not resolved due to local inhomogeneities in the image intensity). When moving away from this reference slice (i.e. to slices n+1 and n+4), fewer control points are identified (if the same threshold as in the reference slice is used) due to further varying image intensity. By subtracting a blurred version of the image (unsharp masking, step b) the intensity inhomogeneities are removed from each image. Applying an image‐adapted threshold, which accounts for overall variations in pixel intensity between slices (step c), leads to accurate identification of all control points present in the images, as depicted in the right column of images in Fig. 3. Figure 4 shows a typical distribution of image intensity inhomogeneity artifacts removed by applying unsharp masking. The image profile is darker at the center and brighter at the top and bottom edges.

### D. Determination of the 3D distortion field

Once the MR and CT 3D matrices of control points are rigidly registered into the same system of coordinates, the 3D distortion field characterized by the local displacement vectors between the image (MR) and world (CT) coordinates is determined. The one‐to‐one correspondence between CT and MR control points is accomplished by first considering the undistorted distribution of CT points as being the reference template. Then the corresponding MR control points are determined in a small region centered on each of the CT control points. This analysis was performed slice‐by‐slice for the transverse, sagittal, and coronal datasets.

The following preliminary steps were performed prior to determining the distortion field:

(1) Cleaning the data: from the MR image processing stage, residual data (e.g. air bubbles) can generate artifacts, specifically additional control points (<1% of all control points) that would alter the accuracy of the distortion values. However, this data can be easily filtered out by applying a third order polynomial fitting technique. Several studies^(^ ^17^ ^,^ ^18^ ^)^ aimed to model the distortion field by using 3rd order polynomials. However, we found that the 3D distortion field exhibits a complex local behavior and can not be accurately described by using such methods. As a first step, we do use the 3rd order polynomials to fit the MR data from each image slice to its corresponding CT data. This polynomial approximates the world coordinates of the control points, and then serves to identify and eliminate the control points due to artifacts, which lie too far away from the control points defined by the polynomials.
(2) Removing the effects of main field ${(B}_{0})$ inhomogeneities: the coordinates of each MR control point, corrected for $B_{0}$ inhomogeneities, are given by the average of its location in the corresponding forward and reverse gradient acquired datasets as follows:
  (1) $$\begin{array}{l} x^{i}=(x_{\text{for}\text{war}d}^{i}+x_{\text{rev}\text{ers}e}^{i})/2\\ y^{i}=(y_{\text{for}\text{war}d}^{i}+y_{\text{rev}\text{ers}e}^{i})/2,\;i=\bar{1,N}\\ z^{i}=(z_{\text{for}\text{war}d}^{i}+z_{\text{rev}\text{ers}e}^{i})/2 \end{array}$$
  where *N* is the maximum number of control points.

The local distortion along each axis is determined as the difference between the coordinates of each corresponding MR and CT control point in their common system of reference:

(2) $$\begin{array}{l} \delta x^{i}=x_{MR}^{i}-x_{CT}^{i}\\ \delta y^{i}=y_{MR}^{i}-y_{CT}^{i}\;\;\;,\;\;i=\bar{1,N}\\ \delta z^{i}=z_{MR}^{i}-z_{CT}^{i} \end{array}$$

The total distortion corresponding to each control point is given by:

(3) $$\delta r^{i}=\sqrt{{\left(\delta x^{i}\right)}^{2}+{\left(\delta y^{i}\right)}^{2}+{\left(\delta z^{i}\right)}^{2}}\;\;\;,\;\;i=\bar{1,N}$$

Applying Eqs. (2) and (3) to all three orthogonal datasets (CT and MR), we can find a double estimate of the distortion field along each axis:^(10)^ (a) *x* and *y* distortion from the scans where the grid sheets were oriented along the transverse plane $\left(\delta x_{\text{tra}},\;\delta y_{\text{tra}}\right)$, (b) *y* and *z* distortion from the scans where the grid sheets were oriented along the sagittal plane $\left(\delta y_{\text{sag}},\;\delta z_{\text{sag}}\right)$, and (c) *x* and *z* distortion from the scans where the grid sheets were oriented along the coronal plane $\left(\delta x_{\text{cor}},\;\delta z_{\text{cor}}\right)$. Specifically, the distortion values along the main axes for the ith control point are given by:

(4) $$\begin{array}{l} \delta x_{0}^{i}=(\delta x_{\text{tra}}^{i}+\delta x_{\text{cor}}^{i})/2\\ \delta y_{0}^{i}=(\delta y_{\text{tra}}^{i}+\delta y_{\text{sag}}^{i})/2,\;\;\;\;i=\bar{1,N}\\ \delta z_{0}^{i}=(\delta z_{\text{sag}}^{i}+\delta z_{\text{cor}}^{i})/2 \end{array}$$

where *N* is the number of control points used to determine the distortion field. Thus from each of the three measured datasets (grid sheets oriented along the transverse, sagittal, and coronal plane, respectively), we assess the in‐plane distortion values with regard to the plane orientation, and evaluate the distortion in the third direction from an orthogonal dataset. However, these quantities represent only an approximation due to the possibility of through‐plane distortion. For each control point, the evaluation of distortion along one particular axis is altered by the uncertainty in determining the distortion values obtained from the datasets acquired in the other two orientations. If, for example, the grid planes are oriented in the transverse plane, the *x* and *y* distortion values can be obtained for a given MR image slice. However, the *z* value (DICOM coordinate of the image slice) assigned by the scanner to that slice might not be the physically correct one, because of possible distortion in the *z* direction. We therefore employed an iterative process to determine the *true* values of 3D distortion associated with each MR control point.

**Figure 5:**
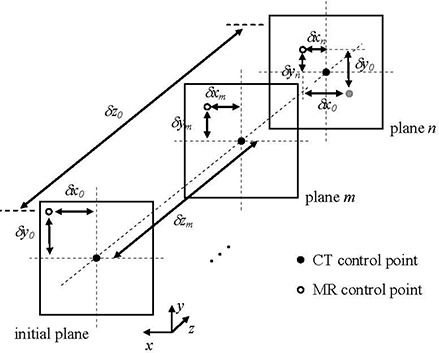
Diagram describing the iteration process for one control point. The distortion values corresponding to the MR control point (hollow dot) may vary depending on its *z* location. Using the initial estimates of distortion given by Eq.(5), we might end up over‐correcting the position of the MR point. Specifically, the location of the MR point would be given by the gray dot (not the CT point or black dot). In our data sets, spacing between subsequent image slices (here in *z* direction) was 1 mm.

To stress the need for an iteration process, the case of only one measured control point (the coordinates determined as the center of mass of each dot area, noted in Section C, step g) in the transverse reconstructed dataset is considered (Fig. 5). If the MR control point (hollow dot) is located in the initial plane and has no *z* distortion, then its true (in‐plane) distortion values are given by $\delta x_{0}$ and $\delta y_{0}$. These values are defined with respect to the coordinates of the CT point (black dot). If, however, the position of the MR control point is also distorted in the *z* direction, the values obtained initially for its in‐plane distortion might not be the true ones because they depend on the *z* coordinate of the plane at which they are measured: $\left(\delta x_{0},\;\delta y_{0}\right)$, $\left(\delta x_{m},\;\delta y_{m}\right)$ and $(\delta x_{n}$, $\delta y_{n}$) are not necessarily identical. Therefore, $\delta x_{0}$ is only an initial estimate and possibly needs to be recalculated in a different image plane (*z* coordinate), thus taking into account distortion in *z* direction. For example, by correcting the *z* coordinate for $\delta x_{0}$ in Fig. 5, the *x* and *y* distortions now need to be calculated on plane *n* and will likely be different from the ones in the initial plane $\left(\delta x_{n},\;\delta y_{n}\right)$. If we had not recalculated the *x* and *y* distortion in plane *n* and applied the values ($\delta x_{0}$, $\delta y_{0}$) found in the initial plane to the MR point's position in *x* and *y* direction in plane *n*, then its new location would be incorrectly given by the gray dot instead of by the black dot in plane *n* in Fig. 5. The error in the distortion correction applied would then be $\left(\delta x_{n}–\delta x_{0},\;\delta y_{n}–\delta y_{0}\right)$. In the same manner, the new distortion values for *x* and *y* $\left(\delta x_{n},\;\delta y_{n}\right)$ will affect the corrections that result for the distortion in the (*y,z*) and (*x,z*) planes, respectively.

The iterative process we developed in order to find the 3D distortion values is applied to each MR control point. For the ith control point defined by the coordinates ${(x}^{i}{,y}^{i}{,z}^{i})$, the initial estimate of distortion is given by $\left(\delta x_{0}^{i},\delta y_{0}^{i},\delta z_{0}^{i}\right)$ from Eq. (4). The iteration process starts with the *x* coordinate. To find ${\delta x}_{1}^{i}$, the new estimate of *x* distortion, we account for the initially found distortions in *y* and *z* by applying the ${\delta x}_{0}^{i}$ and ${\delta x}_{1}^{i}$ shifts to the ith control point. These shifts generally move the control point to a location in between the measured control points’ coordinates. Therefore, ${\delta x}_{1}^{i}$ is determined at the location ${(x}^{i}{,y}^{i}+\delta y_{0}^{i},z+\delta z_{0}^{i})$ by interpolation of the *x* distortion values of neighboring measured control points. In general, the *x* distortion value in the nth iteration is a function of the *y* and *z* distortions determined in the (n‐1)th iteration:

(5a) $$\delta x_{n}^{i}=\delta x_{n}^{i}(x^{i},y^{i}+\delta y_{n-1}^{i},z^{i}+\delta z_{n-1}^{i}),\;\;\;i=\bar{1,N},\;\;\;\;n>0$$

where *n* is the iteration index. The value of ${\delta x}_{1}^{i}$ is subsequently used to estimate in turn the distortions along the *y* and *z* axes. Specifically, the new estimate of *y* distortion (${\delta x}_{1}^{i}$) is determined using a similar relation as Eq. (5a) after applying the ${\delta x}_{1}^{i}$ and ${\delta x}_{0}^{i}$ correction shifts: ${\delta x}_{1}^{i}{=\delta y}_{1}^{i}{(x}^{i}{+\delta x}_{1}^{i}{,y}^{i}{,z+\delta z}_{0}^{i})$ or in general

(5b) $$\delta y_{n}^{i}=\delta y_{n}^{i}(x^{i}+\delta x_{n}^{i},y^{i},z^{i}+\delta z_{n-1}^{i}),\;\;\;i=\bar{1,N},\;\;\;n>0$$

Similarly, the *z* distortion $\delta z_{1}^{i}$ is evaluated at the location corrected for $\delta z_{1}^{i}$ and $\delta y_{1}^{i}$ as ${\delta z}_{1}^{i}{=\delta z}_{1}^{i}{(x}^{i}{+\delta x}_{1}^{i}{,y}^{i}{,+\delta y}_{1}^{i}z)$ or in general

(5c) $$\delta z_{n}^{i}=\delta z_{n}^{i}(x^{i}+\delta x_{n}^{i},y^{i}+\delta y_{n}^{i},z^{i}),\;\;i=\bar{1,N},\;\;\;n>0$$

Once the first iteration values $\left(\delta x_{1}^{i}{,\delta y}_{1}^{i}{,\delta z}_{1}^{i}\right)$ are calculated, the need for a further iteration is determined by using

(6) $$\begin{array}{l} \left|\delta x_{n}^{i}-\delta x_{n-1}^{i}\right|<\varphi \\ \left|\delta y_{n}^{i}-\delta y_{n-1}^{i}\right|<\varphi ,\;\;\;i=\bar{1,N},\;\;\;n>0.\\ \left|\delta z_{n}^{i}-\delta z_{n-1}^{i}\right|<\varphi \end{array}$$

where ϕ is the iteration cutoff threshold. If at least one of the conditions in Eq. (6) is not fulfilled the process initiates an additional iteration. The process stops when conditions (6) are satisfied simultaneously. The final $\left(\delta x^{i},\;\delta y^{i},\;\delta z^{i}\right)$ distortion values are given by Eq. (5), using the shifts determined in the last iteration. The convergence of the iteration process is discussed in Section III. Once the distortions of each control point are determined, distortion maps can be generated at any location in the volume using interpolation methods (spline interpolation, in our case). To validate our distortion correction method, the residual geometric distortions were determined (the local distortion values after rerunning the distortion correction algorithm by using the rectified images as input) (see below).

### E. Image correction

Once the distortion values along each axis (δx, δy, δz) are found, the MR images are corrected by applying spatial and pixel intensity interpolations. To accurately account for the local variation of the 3D distortion field we used a spline transformation. In the process of correcting patient images we have to consider that the center of the images (when data is acquired) does not necessarily coincide with the isocenter of the scanner. Therefore, we first determine the shift between the two before generating distortion maps. This can be found, for our 3 T Intera scanner, from the DICOM header of the patient dataset as follows:

(7) $$\begin{array}{l} x_{\text{shi}ft}^{\text{iso}}=\frac{\text{FOV}}{2}-x_{\text{DIC}OM}\\ y_{\text{shi}ft}^{\text{iso}}=\frac{\text{FOV}}{2}-y_{\text{DIC}OM} \end{array}$$

where $x_{\text{shift}}^{\text{iso}}$ and $y_{\text{shift}}^{\text{iso}}$ represent the shift values in *x* and *y*, FOV is the field of view used to acquire the dataset, and $x_{\text{DICOM}}$ and $y_{\text{DICOM}}$ are the *x* and *y* coordinates obtained from the DICOM header.

## III. RESULTS & DISCUSSION

The phantom MR image intensity‐related artifacts were overcome by applying a technique based on a combination of an unsharp masking with adaptive thresholding that was applied to all images in the 3D datasets. This is critical for automatic and accurate determination of all control points corresponding to each image. In contrast, by choosing a unique threshold to be applied to the entire dataset, the number of control points retrieved automatically is a maximum for slices close to the one used to define the threshold and decreases with increasing distance from the defining slice (Fig. 3). Further manual identification of control points is required, which is labor intensive and can introduce additional user‐related errors. In particular, the method developed by Wang et al.^(9)^ may be sensitive to the number of control points resolved in each image. Specifically, the magnitude of distortion along the *z* axis is strongly correlated to the ability of detecting the control points in all images corresponding to each grid‐oil interface. Images were generated by determining the first derivative along *z* axis and convolved with a cross mask to highlight the grid intersection points. At this point, a certain intensity threshold is selected. The newly generated images contain regions of interest that embed the control points’ relative coordinates. These images are used to obtain an initial estimate of the location of the control points by using 1D profiles along the *x* and *y* axes. Not correcting for local image intensity inhomogeneities and intensity variations across the 3D volume (by using a unique threshold) can result in missing control points that might lead to a misrepresentation of the distortion values. Doran et al.^(10)^ also applied a unique image intensity level to isolate pixel clusters that embed the location of the control points on a 2D grid.

The oil‐filled phantom used in our measurements may contain air bubbles that can misrepresent the actual dot area (i.e. air bubbles next to grid points) and can also generate false control points in between subsequent grid points (air bubbles in the mineral oil). This is a common problem with fluid‐filled phantoms. Techniques based only on applying a threshold to resolve the control points are likely to produce additional (artifactual) control points that can corrupt the accuracy of the distortion maps. The approach we implemented is insensitive to the artifacts related to the presence of air bubbles as it eliminates any image data except the areas located at the intersection of the orthogonal grid lines. Over the entire volume of interest (VOI) analyzed – $260\times 260\times 240\;{\text{mm}}^{3}$ – the CT and MR control points can be identified by our algorithm within one voxel $\left(0.9\times 0.9\times 1\;{\text{mm}}^{3}\right)$ from their world coordinates. This was determined by overlaying the control points on the raw images.

The 3D distortion field was determined by using the iteration process described in Section II. By comparison, the method used by Doran et al. represents the first order approximation of the distortion field. Specifically, the distortion of each MR control point along the main axes was determined as the average value of the distortions measured from two orthogonal datasets as given by Eq. (4). In our technique, the values determined by Eq. (4) are used as a starting point of the iteration process (see Section II).

**Figure 6:**
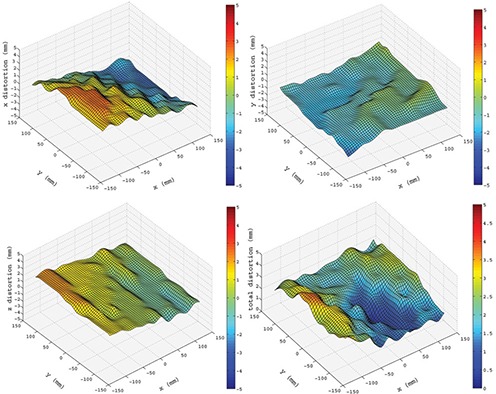
Typical distortion distributions (z=87 mm) for a) δx, b) δy, c) δz, and d) δr. Note that the total distortion plot (d) has a different color bar scale.

**Figure 7:**
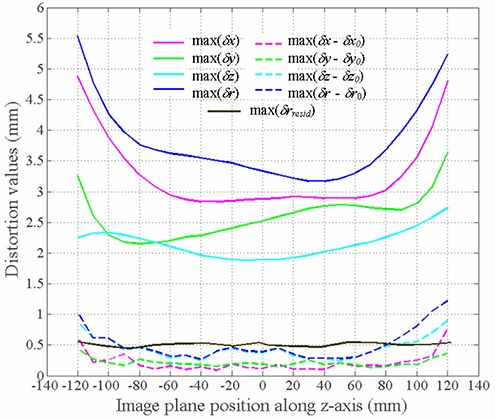
Plot of the distortion values as a function of the image plane location along the *z* axis. The solid lines give the maximum absolute distortion along the main axes (max(δx),max(δy),max(δz). The effect of our distortion correction procedure is evident by comparing the total maximum distortion before and after applying the distortion correction $\left(\max(\delta r),\;\max(\delta r_{\text{resid}}\right)$. The dashed lines show the advantage of our iteratively found distortion values over the ones found by using only an initial estimation of the distortion according to Eq. (4). The absolute difference between the two methods is displayed.

Sample distortion maps corresponding to the image plane located at z=87 mm from the isocenter are displayed in Fig. 6. Figure 7 shows as solid lines the maximum absolute distortion in each image plane along the *z* axis. For all curves, the distortion values increased towards the edges of the FOV. The curves corresponding to the total maximum distortion before and after applying the distortion correction $\left(\text{max}(\delta r)\;\text{and}\;\text{max}(\delta r_{\text{resid}})\right)$ are also shown. The maximum distortion in the $260\times 260\times 240\;{\text{mm}}^{3}$ volume was found to be 5.55 mm. After correcting the images, the maximum residual distortion was determined to be 0.55 mm, which is less than the image voxel size $\left(0.9\times 0.9\times 1.0\;{\text{mm}}^{3}\right)$. A detailed summary of statistical data (mean (*μ*), standard deviation (σ) and maximum (*max*)) of the distortion found and the residual distortion is presented in Table 1.

**Table 1:** Summary of statistical data.

		*Distortion Found for MR Dataset*			*Residual Distortion After Correcting MR Dataset*		
*Distribution Correction Method*	*Axis*	*Mean μ (mm)*	*Standard Deviation σ (mm)*	*Max (mm)*	*Mean μ (mm)*	*Standard Deviation σ (mm)*	*Max (mm)*
$1^{\text{st}}$ Order Estimation (Doran et al.)	x	1.45	0.43	5.84	0.15	0.08	0.96
	y	1.13	0.34	4.49	0.91	0.04	0.53
	z	1.10	0.13	3.55	0.21	0.09	1.22
	r	2.25	0.56	6.70	0.25	0.14	1.54
	x	1.31	0.32	5.07	0.07	0.03	0.36
Iterative Process	y	0.76	0.26	4.05	0.04	0.02	0.20
(Our Work)	z	0.76	0.07	2.65	0.07	0.03	0.38
	r	1.86	0.41	5.55	0.14	0.05	0.55

In Fig. 7, the dashed lines represent the difference between the maximum distortion along the main axes obtained with our iterative method and the first order approximation.^(10)^ The maximum difference was found to be about 1.2 mm. Furthermore, the data in Table 1 shows that the residual distortion after applying only the first order approximation is 1.54 mm, which is significantly greater than the image voxel size. This implies that the iterative method needs to be applied for a more accurate characterization of the 3D distortion field. It should be noted that the iterative method shows its greatest advantage compared to the method of Doran et al. at the edges of the FOV. It can be reasonably expected that this advantage is even more pronounced for larger volumes (e.g. prostate studies) because the accuracy of the initial distortion estimate decreases as the distortion field gradient increases with distance from isocenter^(10)^ and the correction of through plane distortion (with regard to each orthogonal plane) becomes more important. We are planning to investigate this issue further by using a new, wider phantom that can sample the MRI bore more completely and eventually allow the MRI‐based RTP of larger volumes.

With the specific MRI sequence used in this study, the iteration process is required to correct the raw image data for the regions located beyond z=100mm and z=−115mm; otherwise the residual distortion would exceed the size of one voxel. Since most radiation treatment plans extend beyond these *z* values, iterative distortion correction should be employed, especially because the difference between iterative and noniterative distortion correction shows an increasing trend towards the edges for the FOV sampled in this study (Fig. 7). Furthermore, patients are not always centered around the isocenter (see Eq. (8)) and shifts of a few cm can easily occur. Even when imaging a relatively small volume (e.g. sphere with 20 cm diameter for a typical brain study), a larger volume needs to be accurately mapped to provide enough information to correct for distortion in the patient datasets.

Regarding the behavior of our iteration process, it converges due to a slow local variation of the distortion values (δx,δy,δz) corresponding to each control point. With every iteration step, the difference between two consecutively determined distortion values decreases until this difference is comparable to the iteration cutoff threshold, which was chosen to minimize the residual distortion (i.e. φ=0.2 mm). We found that a further reduction in φ does not result in an improvement of the overall residual distortion. To detect lack of convergence of our algorithm, we monitor the residual distortion. Lack of convergence is defined as a residual distortion greater than the values obtained from the initial estimation (Eq. (4)). If the iteration results had exceeded those values, the iterative method had failed and the algorithm would then use those initial values as the resulting distortion values. However, this case was never observed in our tests. For our particular case (scanner type, imaging sequence, VOI size, etc.), the maximum number of iterations required for the algorithm to converge was four. Near the periphery of the VOI, depending on the magnitude of local distortions used in the iteration process, data is required from outside the mapped field (VOI). This data can be easily obtained through interpolation (in our case ‐ spline interpolation). Therefore, in these regions, the algorithm does not diverge due to lack of information. The outcome of the iteration process is not significantly dependent on the spatial coordinate used as a starting step (*x*, *y* or *z*); the maximum difference between different scenarios (e.g. *x*, *y* or *z* first) was within 2%.

To verify the reproducibility of the distortion field distribution we repeated the procedure, including phantom setup and image analysis, three times. We found that the overall discrepancy was within 0.2 mm. This level of accuracy was guaranteed by the use of the alignment jig and the ability of our image processing algorithm to resolve the control points’ spatial location.

**Figure 8:**
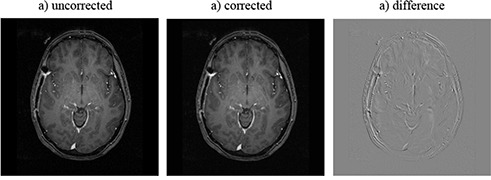
A sample MR image slice of a radiation therapy patient's brain (patient was diagnosed with GBM): a) raw image, b) same image slice corrected using the distortion map determined using our procedure, and c) the difference of the two.

The maximum total distortion for a typical brain study VOI – 10 cm radius – was found to be about 4 mm, suggesting that the MR brain images need to be corrected before being used in the treatment planning process. Figure 8 shows sample MR image slices of a radiation therapy GBM patient: the raw image acquired from the scanner, the same image slice corrected using the distortion map, and the difference between the uncorrected and corrected images. The distortion correction method was implemented as a necessary step in our MRI‐based treatment planning procedure.^(^ ^1^ ^,^ ^2^ ^)^

Wang et al. also investigated VOIs relevant to brain studies and claimed a mean residual error in the measured control points’ coordinates as being between 0.1 and 0.2 mm. We obtained a similar value of 0.14 mm by applying our method. In both cases, measured distortion is below the pixel resolution. However, it is difficult to compare the details of our method with the work of Wang et al. as the latter study did not address aspects typically present in a distortion correction process such as: (a) the process of generating the world coordinates for the 3D distribution of the control points, (b) the registration of corresponding world and image coordinates of the control points, and (c) the correction of MR image intensity artifacts.

Doran et al. used a phantom built from three orthogonal interpenetrating arrays of water‐filled tubes placed in an empty box. One possible limitation of this design is that the tube‐air interface might generate large susceptibility effects leading to an altered accuracy in determining the distortion field. Regarding the control point identification process, the reference 3D matrix of CT control points was generated manually. This is a laborious task from a routine quality assurance perspective, especially when using a large number of control points (approx. 10,000) to maximize accuracy. Manual identification of the control points might also introduce user‐related errors. Furthermore, Doran et al. determined the distortion along each axis as being the average of distortion values estimated from two orthogonal planes. As discussed in Section II, this approach represents only an approximation of the distortion values because the mutual interaction among the distortions along each spatial dimension is not accounted for. An iteration process would be needed for a complete quantification of the 3D distortion field. Also, another limitation of the work conducted by Doran et al. is that the authors performed the analysis on image data with a voxel resolution of $1.88\times 1.88\times 5\;{\text{mm}}^{3}$. As a result, the resolution in the slice selection direction was quite limited, making the evaluation of the distortion field difficult. In our case, the data was acquired with a resolution of $0.9\times 0.9\times 1\;{\text{mm}}^{3}$. We expect that our analysis is more accurate than the one by Doran et al.

The novelty of the method developed here consists of:

(a) adaptive technique for the accurate and automatic identification and extraction of the control points’ location by compensating for MR image intensity inhomogeneities
(b) data cleaning tool based on polynomial fitting that facilitates the automatic registration of the CT and MR control points
(c) iteration process required to determine the 3D distortion field when applied to methods^(10)^ that incorporate multiple orthogonal 2D distortion datasets.

Our method can be implemented by using either grid sheets or rod‐type phantoms (the most common designs), unlike other approaches that are dependent on the phantom type.^(9)^ This would allow the use of the most cost‐efficient phantom design. In the case of a grid sheets‐based design (used in this work), three scans are required due to the phantom's limited 2D symmetry. However, in the case of the 3D rod type phantom,^(10)^ one scan is enough to acquire all the data. Considering that the system‐related and object‐induced distortions are analyzed independently using different approaches, the system‐related distortion correction method presented in this study can be used in conjunction with any object‐induced distortion correction technique available in the literature^(^ ^8^ ^,^ ^12^ ^)^ to correct patient images.

## IV. CONCLUSIONS

We developed a technique to determine scanner‐induced geometric distortions that consists of two key components: an adaptive control points’ identification and registration tool, and an iterative algorithm that calculates the best estimate of 3D distortion.

It was found that over a volume of $260\times 260\times 240\;{\text{mm}}^{3}$, the 3D distortions can be successfully mapped to within the voxel resolution of the raw imaging data. Namely, the total distortion was found to be 5.55 mm with a μ and σ of 1.86 mm and 0.41, respectively. After applying the image correction algorithm the residual distortions were 0.55 mm/0.14 mm/0.05 mm, respectively. The iterative approach taken in this work becomes increasingly important as the FOV is widened.
